# Embolization of jugular bulb diverticula in the management of pulsatile tinnitus

**DOI:** 10.3389/fneur.2026.1951324

**Published:** 2026-10-08

**Authors:** Timothy White, Cassidy Werner, Brianna Donnelly, Varun Sridhar, Kadir Ozler, Justin Turpin, Kelly Hannsgen, Shyle Mehta, Athos Patsalides

**Affiliations:** 1 Northwell Health, New Hyde Park, NY, United States; 2 Donald and Barbara Zucker School of Medicine at Hofstra/Northwell, Hempstead, NY, United States

**Keywords:** coil embolization, jugular bulb diverticula, pulsatile tinnitus, venous sinus, venous sinus stenting

## Abstract

**Introduction:**

Pulsatile tinnitus (PT) caused by a high riding jugular bulb or prominent diverticulum can negatively impact quality of life. This study evaluates the effectiveness of endovascular treatments, including stenting, coiling, and Woven EndoBridge (WEB; Terumo Neuro, Aliso Viejo, CA) devices, for managing jugular bulb diverticula (JBD)-induced PT.

**Methods:**

A retrospective review was conducted from September 1, 2020 to April 30, 2026, identifying twelve patients diagnosed with JBD. Data extracted included demographics, PT characteristics, treatment details, and outcomes. Endovascular interventions were pursued after in depth work-up, often including balloon test occlusion. The outcomes and techniques of various interventions are described.

**Results:**

Twelve patients (eight females and four males) underwent endovascular treatment for JBD, with a median age of 40.5 years and a median BMI of 25.8 kg/m^2^. Balloon-test occlusion (BTO) showed at least moderate improvement in 7/8 patients (87.5%). Stent-assisted coiling for the JBD was performed in eleven cases (91.7%), and placement of a WEB was successful in one patient. There were two unsuccessful attempts in placing a WEB device, and treatment was converted to stent-assisted coiling. At an average last follow-up of 7.25 months, nine patients (75%) had complete resolution and three (25%) had significant improvement (25%) of their symptoms.

**Conclusion:**

Endovascular interventions are safe and effective for treating JBD-related PT. BTO is an important intraprocedural tool that may help predict treatment success. Further investigation into optimal patient selection and the role of specific devices in treating JBD is warranted.

## Introduction

Pulsatile tinnitus (PT), a subtype of tinnitus that is often synchronized with the patient's heartbeat, can significantly worsen quality of life (1). Unlike non-pulsatile tinnitus, which is a continuous ringing, PT typically stems from vascular anomalies. One series found that venous sinus stenosis was the cause of PT in 34% of patients that underwent evaluation. Other vascular causes include dural arteriovenous fistulae, or, less often, from the presence of a jugular bulb diverticulum (JBD), which is an outpouching of the jugular bulb that can

lead to turbulent blood flow and subsequent PT (2). Abnormal dilations in this area can compress or transmit pulsations from turbulent flow to adjacent structures including the cochlea and vestibular apparatus causing an auditory disturbances (3). The management of JBD-related PT has evolved over time, with conservative measures often providing insufficient relief and surgical approaches involving invasive skull base procedures which can have significant morbidity (3). The advent of endovascular techniques has introduced novel therapeutic options as well as challenges (4).

Stenting and stent-assisted coiling normalizes venous outflow by reinforcing the vessel wall and reducing the turbulent flow that causes PT (4). This minimally invasive approach has shown promising results, offering symptomatic relief and improved quality of life with low complication rates (4, 5). Although sigmoid sinus diverticulum treatment is increasingly common, the treatment of jugular bulb diverticula is still rare and technically complex. For these reasons, the treatment of JBD in patients with PT is not well represented in the literature. Here, we present our series of patients with PT who underwent endovascular obliteration of a JBD.

## Methods

Our study was approved by a local institutional review board at our institution. We conducted a retrospective search of our venous sinus stenting (VSS) database between September 1, 2020 and April 30, 2026 to identify patients ≥18 years of age who presented with pulsatile tinnitus, were diagnosed with JBD on imaging, and met at least one of the following three requirements: (a) if PT symptoms were present in the angiography suite during time of intervention and BTO was performed, at least some resolution of PT occurred during balloon inflation, (b) a provocative saline injection test into the jugular bulb dome reproduced PT symptomology, or (c) a failure of prior conservative options facilitated a discussion between the interventionalist and patient regarding the imaging findings of a JBD and the debilitating nature of the patient's PT, resulting in the patients opting for endovascular treatment. Exclusion criteria included patients who presented with PT and were diagnosed with JBD on imaging but did not experience any resolution of PT if BTO was performed, as endovascular treatment would not have been beneficial. Variables extracted for analysis were age, sex, symptoms, acetazolamide (Diamox) dose, narcotic dose, BMI, race, lumbar puncture opening pressure, technical details of stenting, follow-up results, and retreatment rate. Written informed consent was obtained from the individuals for the publication of any potentially identifiable images or data included in this article.

### Pulsatile tinnitus evaluation including balloon test occlusion

After presenting with PT, patients are referred for a consultation with an otolaryngologist for the assessment of underlying otological causes of PT. As part of our protocol, all patients are ordered for a contrast-enhanced MRV of the head to evaluate for venous sinus abnormalities and non-contrast MRA of the head and neck to rule out the presence of dural arteriovenous fistula or arteriovenous malformation of the head and neck for initial imaging. We do not routinely order contrast-enhanced non-vascular MR sequences on our patients unless there is suspicion for intraparenchymal etiology. Contrast-enhanced CT is sometimes obtained to assess if a jugular bulb diverticulum is present due to inconclusive MRV findings.

To aid in our diagnosis of vascular PT, we utilize neck compression and assess for changes in tinnitus intensity, as prior literature has shown 90% specificity for venous sinus stenosis as a cause of PT (5). In our experience, this remains a useful diagnostic feature, even in cases of PT caused by JBD. When exam findings were compatible for vascular causes of PT, and a magnetic resonance imaging or angiography excluded a shunting lesion, the patients underwent a diagnostic venogram. When possible, patients were given noise-canceling headphones and asked to rate their PT on a scale of 1 to 10 before and after a balloon test occlusion (BTO) across the jugular bulb prior to pursuing any endovascular treatment.

Venous manometry was performed with a 0.027 inch microcatheter from the superior sagittal sinus down the venous outflow tract if there was concern for possible venous sinus stenosis. If no pressure gradient of 4 mmHg or greater was found across the site of suspected stenosis during manometry, a BTO was performed. The BTO was performed using either Eclipse dual lumen balloon catheters (Balt, Irvine, CA) or a single Walrus balloon-guide catheter (Q'Apel, Fremont, CA). Initially, two Eclipse 6 mm × 20 mm balloons were used; however, the workflow was changed to utilize a Walrus balloon-guide catheter (Q'Apel, Fremont, CA) given its ability to fit into and occlude the entirety of the jugular bulb. The balloons were positioned in the JBD and inflated under fluoroscopic guidance while the patient was wearing noise canceling headphones. This was only performed if the patient was able to hear the pulsatile tinnitus prior to balloon inflation. Care was taken to not tell the patient when the balloon was inflated or deflated. Once the balloon was inflated, venography was performed to confirm occlusion of the JBD. At least three inflations and deflations were performed while asking the patient to rate the PT on a scale from 0 to 10. In accordance with previous literature using qualitative descriptions of changes in PT severity for BTO, patients who experienced at least a mild improvement in PT following BTO were considered a candidate for endovascular treatment (6, 7). All patients were placed on dual antiplatelet therapy (Aspirin 325 mg and Plavix 75 mg) 5 days prior to the procedures, so that if the BTO was positive, patients were prepared for venous sinus stent placement. In some patients, Woven EndoBridge (WEB; Terumo Neuro, Alisa Viejo, CA) placement was attempted. WEB placement was done with the patients awake while stenting was performed under general anesthesia. After the procedure, patients remained on dual antiplatelet therapy for 3 months, at which point Plavix was discontinued and the patient remained on aspirin monotherapy for the nine additional months.

### Statistical analysis

Continuous variables were reported as means and standard deviations or medians and interquartile range according to data distribution. Categorical variables were reported as absolute numbers and percentages. Statistical analyses were performed using Microsoft Excel.

## Results

### Patient demographics and presenting symptomatology

During the study period, there were 14 total patients with PT diagnosed with JBD at our institution. Two JBD patients did not experience any resolution of PT during BTO, so the decision was made to stop the procedure and proceed with conservative treatment instead, and they were excluded from the study. Twelve patients (eight females and four males) underwent endovascular treatment of JBD. The median age was 40.5 years (range 23–58 years; Table 1). The median BMI was 25.8 kg/m^2^ (range 17.8 to 39.4 kg/m^2^). Ten patients were Caucasian (83.3%), one was “multiracial” (8.3%), and one was Asian (8.3%; Table 1).

**Table 1:** Baseline patient demographics, symptoms, and past treatments.

Patient ID	Age	Sex	BMI (kg/m^2^)	Ethnicity	Pulsatile tinnitus (R/L/bilateral)	Jugular bulb diverticulum side	Jugular bulb diverticulum pressure (mmHg)	Approximate decrease in PT with BTO (Y/N)	Provocative saline injection testing reproduced PT symptomology	Previous treatments
1	50	F	37.6	Caucasian	Bilateral	L	10	N/A	N/A	Stenting of left transverse-sigmoid junction
2	31	F	25	Caucasian	Bilateral	R	15	N/A	N/A	None
3	45	F	17.8	Caucasian	L	L	8	Slight improvement	Yes	Stenting of left transverse sinus and left transmastoid superior semicircular canal plugging
4	23	F	22.9	Caucasian	Bilateral	R	9	N/A	Yes	None
5	29	M	18.1	Caucasian	L	L	Not reported	80%	N/A	Coiling of L transmastoid emissary vein
6	33	F	32.9	Caucasian	R	R	Not reported	Some improvement	N/A	None
7	48	M	30.1	Asian	R	R	11	Near complete resolution	N/A	None
8	33	M	23.1	Caucasian	R	R	7	100%	Yes	None
9	36	M	19.6	“Multiracial”	R	R	12	N/A	N/A	None
10	52	F	24.1	Caucasian	R	R	Not reported	50% (four out of 10 to two out of 10)	Yes	None
11	58	F	39.35	Caucasian	L	L	4	Some improvement	N/A	None
12	56	F	19.46	Caucasian	R	R	Not reported	100% (five out of 10 to 0 out of 10)	N/A	None

Debilitating PT was the presenting symptom in all patients, with the laterality being right-sided in six patients (50%), left-sided in three patients (25%), and bilateral in three patients (25%). In addition to PT, six patients experienced headaches, two experienced visual disturbances, one patient endorsed numbness of their head, one experienced a feeling of pressure or fullness in their head, one endorsed mild hearing loss, one experienced autophony, and one endorsed dysestheisa in their face and mouth. Three patients (25%) had prior interventions for PT; one patient had VSS of the left transverse-sigmoid junction, one patient had VSS of the left transverse-sigmoid junction as well as left superior semicircular canal plugging, and one patient had coiling of the left transmastoid emissary vein. In all three patients, PT persisted, leading to the need for retreatment to address the JBD. To identify the JBD, contrast-enhanced MRV was performed in 11 patients, one of whom additionally underwent contrast-enhanced CT. Non-contrast MRV was performed in one patient, who subsequently underwent contrast-enhanced CT. JBD was confirmed on the right side in eight patients (66.7%) and on the left side in four patients (33.3%; Table 1).

Eight JBD patients with audible PT symptoms in the angiography suite underwent BTO, all of whom experienced at least a mild improvement in PT following balloon inflation and were therefore considered a strong candidate for endovascular treatment. Two patients were able to rate their PT on a scale of 1–10 before and after balloon inflation. One patient rated their PT a 4 prior to inflation and a 2 after inflation, while the other rated their PT a 5 prior to inflation and 0 after inflation. For the six patients who did not objectively rate their PT before and after BTO, one endorsed “slight improvement,” two endorsed “some improvement,” one achieved “80% improvement,” one described “near complete resolution,” and one described “complete resolution” of PT after balloon inflation. Overall, seven out of eight patients (87.5%) who underwent BTO described at least moderate improvement in PT after balloon inflation. In addition, three patients who underwent BTO subsequently received provocative testing with saline injection which reproduced their PT, providing further evidence that the JBD was responsible for their symptoms and endovascular treatment could be beneficial (Table 1).

Two out of four patients who did not undergo BTO did not hear the PT in the angiography suite even with noise canceling headphones, and therefore BTO was not performed. One of these patients underwent provocative saline injection testing and correctly identified the number of saline injections, so treatment was recommended. For the three patients who did not undergo BTO or provocative saline injection testing, as previously mentioned, one patient had just undergone successful stenting of the left transverse-sigmoid junction, but was still experiencing pulsatile tinnitus postprocedurally, so after discussion with the patient, endovascular treatment of their JBD was carried out. For the remaining two patients, after discussions between the interventionalist and the patient regarding their venogram findings, the significant negative impact of their PT on their quality of life, and failure of prior conservative treatment options, the patients opted for endovascular treatment.

### Endovascular intervention

Five patients had Carotid Wallstents (Boston Scientific, Marlborough, MA) and six patients had Precise Pro stents (Cordis, Hialeah, FL) deployed across the jugular bulb (Table 2). Post-stent angioplasty was performed in five patients (45%) using Sterling (Boston Scientific, Marlborough, MA) or Eclipse balloons all in the setting of Wallstents. Stent lengths ranged from 31 to 41 mm. Coiling of the JBD was performed in all eleven patients who underwent stenting.

**Table 2:** Treatment details and follow-up information.

Patient number	Successful treatment?	Proximal stent site	Distal stent site	Stent 1 diameter/length	Stent 2 diameter/length	Type of stent 1	Type of stent 2	Number of stents	Type of guide catheter	Type of intermediate catheter	Attempted WEB placement?	Coiling?	Post-stent angioplasty?	Length of stay (days)	PT on follow up	Length of follow up (months)
1	Yes	L transverse sinus	L sigmoid sinus	7 × 40mm	n/a	Precise Pro	n/a	1	Ballast	Navien	6 × 20 mm dual-lumen Eclipse balloon advanced into L jugular bulb via a BMX catheter situated in the L internal jugular vein. Placement was successful.	No	No	0	Resolved	15
2	Yes	R sigmoid sinus	Upper segment of R internal jugular vein	Not reported	n/a	Wallstent	n/a	1	Infinity	Navien	WEB inserted with multiple repositionings. The wide neck configuration of the diverticulum precluded stable deployment, so it was removed.	Yes	Sterling 8 × 20 mm balloon	1	Resolved	5
3	Yes	Neck of the L jugular bulb	L sigmoid sinus	10 × 37 mm	n/a	Wallstent	n/a	1	Ballast	Navien	6 × 20 mm dual-lumen Eclipse balloon inserted with WEB, but the position was suboptimal so it was removed.	Yes	No	0	Resolved	23
4	Yes	R sigmoid sinus	Upper segment of R internal jugular vein	10 × 31 mm	n/a	Wallstent	n/a	1	Ballast	Navien	No	Yes	Sterling 8 × 20 mm balloon	1	Improved	1
5	Yes	L sigmoid sinus	L internal jugular vein	10 × 41 mm	n/a	Not reported	n/a	1	Ballast	Navien	No	Yes	Sterling 9 × 40 mm balloon	0	Resolved	11
6	Yes	R sigmoid sinus	Upper segment of R internal jugular vein	10 × 37mm	n/a	Wallstent	n/a	1	Ballast	Navien	No	Yes	Sterling 8 × 20 mm balloon	1	Improved	1
7	Yes	R sigmoid sinus	Upper segment of R internal jugular vein	8x36mm	n/a	Wallstent	n/a	1	Ballast	Esperance	No	Yes	Eclipse 6 × 20 mm dual-lumen balloon	1	Resolved	15
8	Yes	R sigmoid sinus	Upper segment of R jugular vein	10 × 30 mm	10 × 40 mm	Precise Pro	Precise Pro	2	Walrus	Navien	No	Yes	No	0	Improved	4
9	Yes	R sigmoid sinus	Upper segment of R jugular vein	8 × 30 mm	8 × 40 mm	Precise Pro	Precise Pro	2	Ballast	Navien	No	Yes	No	1	Resolved	5
10	Yes	R occipital sinus	Upper segment of R jugular vein	6 × 30 mm	8 × 40 mm	Precise Pro	Precise Pro	2	Ballast	Navien	No	Yes	No	1	Resolved	5
11	Yes	L transverse sinus	Distal sigmoid sinus	7 × 40 mm	8 × 40 mm	Precise Pro	Precise Pro	2	Mega Ballast	Raptor	No	Yes	No	0	Resolved	1
12	Yes	Right sigmoid sinus	Upper segment of R jugular vein	8 × 30 mm	10 × 40 mm	Precise Pro	Precise Pro	2	Ballast	Raptor	No	Yes	No	2	Resolved	1

One patient had a standalone WEB placement, although WEB placement was attempted and unsuccessful in two additional patients. In one of these patients, a 6 × 20 mm dual-lumen Eclipse balloon was inserted with the WEB, but during post procedure angiography, the WEB was found to be displaced and was subsequently retrieved using a snare. In the other patient, the WEB was inserted with multiple repositioning attempts and ultimately the wide neck configuration of the JBD precluded stable positioning.

### Treatment outcomes

The hospital stay post-treatment was no more than two days, with five patients (41.2%) discharged on the same day of treatment, six discharged on post-treatment day one (50%), and one on post-treatment day two (8.3%). There was one patient with a partial transient accessory spinal nerve palsy (could not elevate her arm above the shoulder), which resolved completely in 3 months. There were no additional adverse events.

Of the 12 treated patients, complete resolution of symptoms was achieved in nine patients (75%, Table 3). An additional three patients reported significant improvement or near complete resolution (25%). Of note, one of the three patients with improvement only had a 20% reduction in PT during the BTO. Length of follow-up varied, with a mean duration of 7.25 months (range 1–26 months).

**Table 3:** Summary of imaging characteristics, endovascular treatment strategies, and post-treatment outcomes in patients with jugular bulb diverticula.

Category	Characteristic	Finding
Imaging characteristics	Imaging used to identify JBD	Contrast-enhanced MRV: 11/12; non-contrast MRV: 1/12; CT additionally obtained in 2/12
	JBD laterality	Right: 8/12 (66.7%); left: 4/12 (33.3%)
	Morphologic features relevant to treatment	Wide-neck morphology was specifically described in one patient and precluded stable WEB deployment
Endovascular treatment	Stent-assisted coil embolization	11/12 (91.7%)
	Standalone WEB embolization	1/12 (8.3%)
	WEB device attempts	Three total attempts: one successful; two unsuccessful, with subsequent treatment using stent-assisted coiling
	Post-stent angioplasty	Performed in five patients
Post-treatment outcomes	Complete resolution of pulsatile tinnitus	9/12 (75%)
	Significant or near-complete improvement	3/12 (25%)
	Hospital length of stay	Same-day discharge: 5/12; post-treatment day 1: 6/12; post-treatment day 2: 1/12
	Procedure-related adverse events	One transient partial accessory spinal nerve palsy, with complete resolution by 3 months
	Additional adverse events	None reported

JBD, jugular bulb diverticulum; MRV, magnetic resonance venography; CT, computed tomography; WEB, Woven EndoBridge.

### Case 1 example

A 50-year-old woman presented to the office with a chief complaint of chronic progressive left-sided PT and headache which was most severe in the morning. Pressing on the left side of her neck or head rotation to the left diminished the amplitude of the PT. Magnetic resonance angiography (MRA) revealed no evidence of vascular malformation and MR venography (MRV) revealed bilateral transverse sinus stenosis, more severe on the left with associated sigmoid/jugular bulb enlargement concerning for diverticulum. The MRV revealed left-sided transverse sinus dominance. Neuro-ophthalmology examination was negative for papilledema, reducing the suspicion for concomitant idiopathic intracranial hypertension. She was scheduled for catheter venogram with possible VSS and started on dual antiplatelet therapy 5 days prior.

Under local anesthesia, right common femoral vein access was obtained and an 8F short sheath was inserted and attached to a heparinzed flush line. A 0.088″ Ballast long sheath (Balt, Irvine, CA) was inserted into the left internal jugular vein. The diagnostic catheter and wire were removed and a 0.072″ Navien intermediate catheter (Medtronic, Minneapolis, MN) over an 0.027 ″ Headway 27 microcatheter (Terumo Neuro, Aliso Viejo, CA) and 0.014″ Synchro Select standard microguidewire (Stryker, Portage, MI) was navigated into the superior sagittal sinus (SSS). The microwire was removed and angiography was performed showing a left-side dominant transverse sinus with moderate to severe stenosis at the junction of the left transverse and sigmoid sinuses along with a moderate size left jugular bulb diverticulum (Figure 1a). The microcatheter was then attached to a manometer and revealed a SSS pressure of 22 mm Hg and a trans-stenotic gradient between the proximal transverse and distal sigmoid sinuses of 7 mm Hg. Given the presence of a gradient the patient was placed under general anesthesia with plans for stenting of the transverse sigmoid junction stenosis.

**Figure 1:**
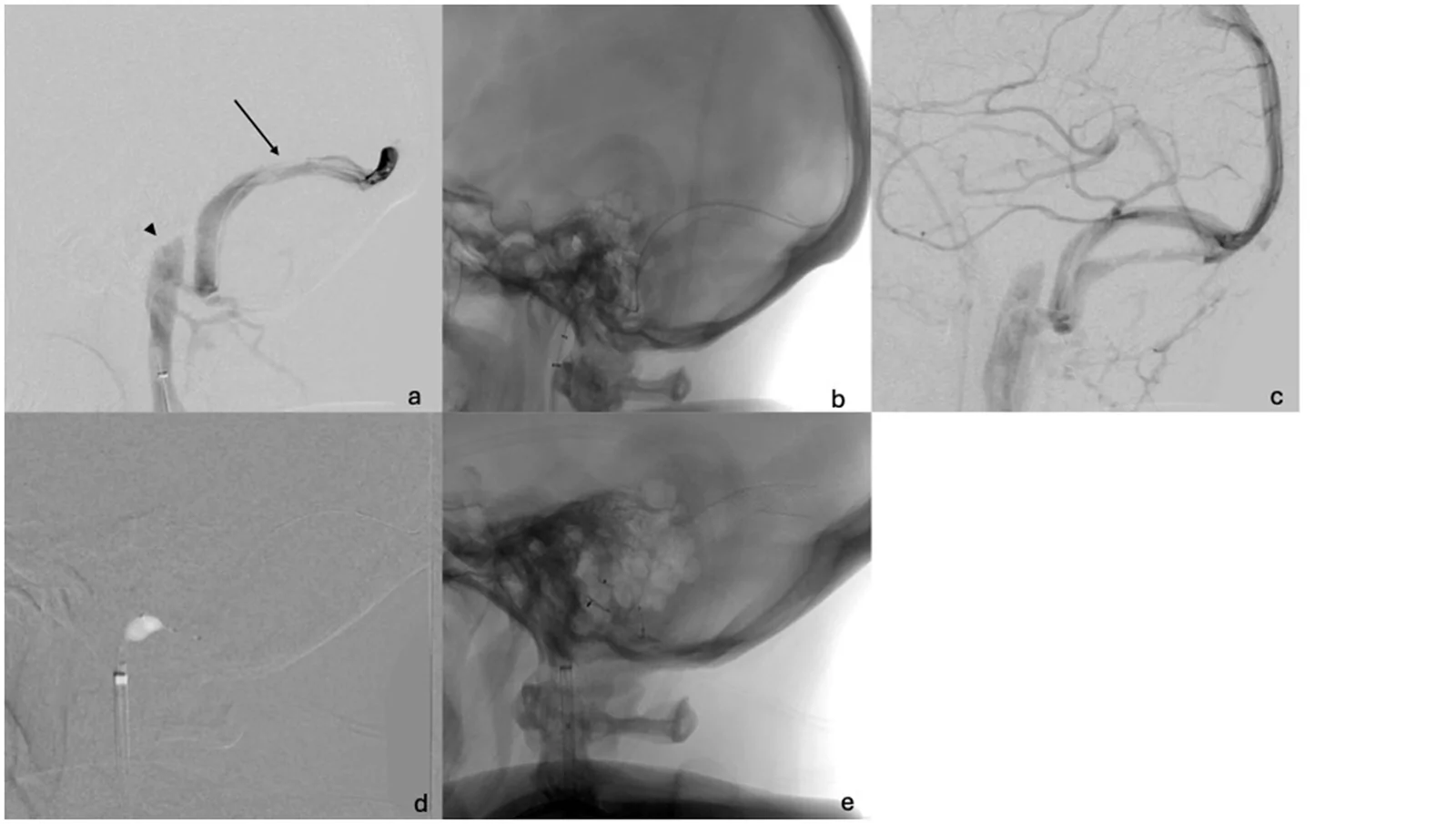
Endovascular treatment of left-sided pulsatile tinnitus with transverse-sigmoid sinus stenting followed by JBD embolization. **(a)** Pre-treatment venography demonstrates a left-dominant transverse sinus with moderate-to-severe stenosis at the transverse-sigmoid junction (arrow) and a moderate-sized left JBD (arrowhead). **(b)** Deployment of a 7 × 40 mm Precise Pro stent across the transverse-sigmoid junction stenosis. **(c)** Post-stent venography demonstrates restoration of normal antegrade venous outflow without residual stenosis or compromise of the vein of Labbe. **(d)** BTO of the left internal jugular vein distal to the JBD using an Eclipse 2L balloon, resulting in complete temporary resolution of pulsatile tinnitus. **(e)** Final venography following deployment of a 10 × 5 mm WEB SL device within the JBD. JBD, jugular bulb diverticulum; BTO, balloon test occlusion.

Under general anesthesia, right radial artery access was obtained and arterial angiography was performed for roadmap guidance. A Precise Pro 7 × 40 mm stent over an exchange-length 0.014 ″ Spartacore wire (Abbott Laboratories, Abbott Park, IL) was deployed across the left transverse-sigmoid junction stenosis (Figure 1b). Post-procedure angiography demonstrated normal anterograde flow into the deep and superficial venous systems without evidence of residual stenosis or impedance of the vein of Labbe (Figure 1c).

The patient was extubated, and she reported continuing to hear the PT. After discussion, we decided to place an intrasaccular WEB device into the JBD which was believed to be the source of residual PT. A 6 × 20 mm Eclipse 2L balloon (Balt, Irvine, CA) was inflated distal to the JBD to occlude jugular outflow and assess for a decrease in PT (Figure 1d). The BTO resulted in a complete resolution of her PT. A 10 mm × 5 mm WEB SL was deployed within the JBD through a 133 cm VIA 33 microcatheter (Terumo Neuro, Aliso Viejo, CA) with the balloon inflated to help position the device within the JBD (Figure 1e). All catheters were removed and closure was obtained with a venous Vascade closure device (Haemonetics. Boston, MA). At 26 month follow-up, her left-sided PT had completely resolved without recurrence.

### Case 2 example

A 31-year-old female presented to the office with severe right-sided PT that had been getting progressively worse and was significantly affecting her quality of life. She described the PT as a constant, whooshing sound that was in sync with her pulse. Pressing on the right side of her neck and turning her head to the right improved the PT. MR vessel imaging revealed no evidence of dural arteriovenous fistula or arteriovenous malformation and demonstrated stenosis at the junction of the right transverse and sigmoid sinus. A CT of her neck revealed a large right jugular bulb diverticulum. She was scheduled for catheter venogram with possible VSS and coil embolization of the jugular bulb diverticulum. She started dual antiplatelet therapy 5 days prior to the procedure.

Under local anesthesia, right common femoral vein access was obtained and an 8F short sheath was inserted and attached to a heparinized flush line. A 0.088″ AXS Infinity LS sheath (Stryker, Portage, MI) was navigated into the right internal jugular vein and angiography was performed of the internal jugular vein. A Headway 27 microcatheter (Terumo Neuro, Alisa Viejo, CA) was then advanced over a Synchro soft microwire into the superior sagittal sinus and angiography was performed, demonstrating a dominant right transverse venous sinus and mild stenosis of the right jugular bulb as well as a prominent jugular bulb diverticulum (Figure 2a). Venous manometry was conducted at different points of the right venous system, from the SSS to the JB; however, only a trans-stenotic gradient of 1 mmHg was observed. A BTO resulted in complete resolution of her PT. Following discussion with the patient regarding the findings, the decision was made to proceed with embolization of the right jugular bulb diverticulum. The patient was intubated and managed with general anesthesia. The right radial artery was accessed for arterial angiography.

**Figure 2:**
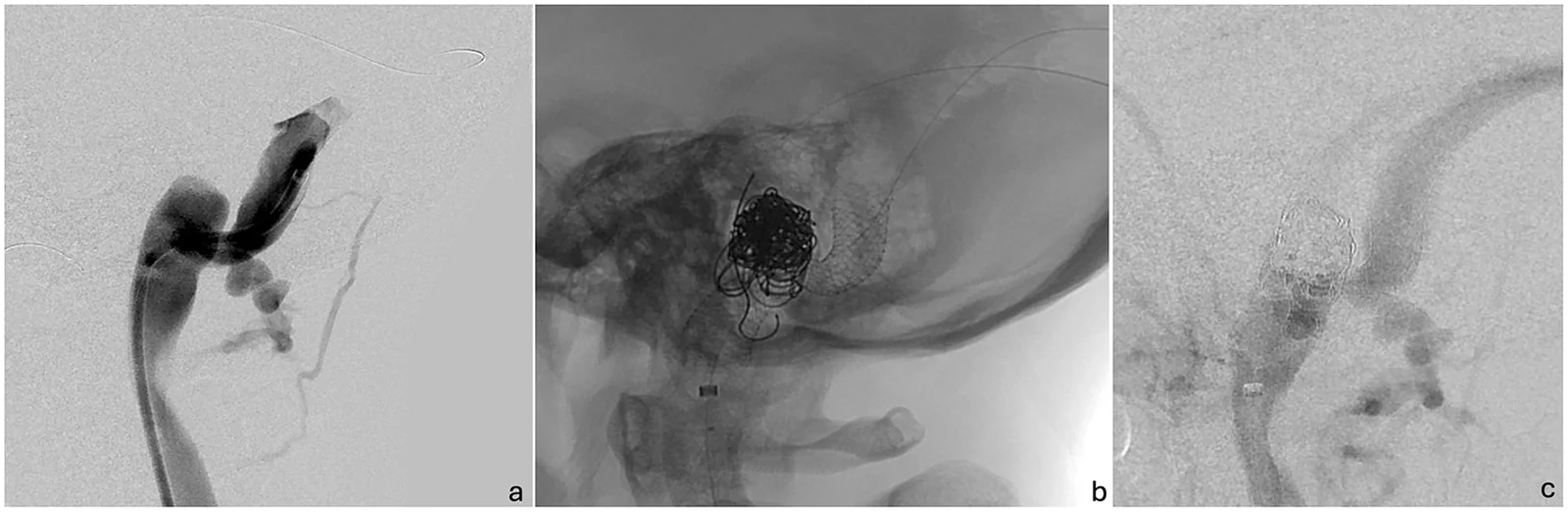
**(a)** Right sigmoid sinus venography showing a right jugular bulb diverticulum in a lateral projection. Post-procedure unsubtracted lateral x-ray **(b)** and subtracted right common carotid artery injection venous phase **(c)** images show patency of the right sigmoid sinus and internal jugular vein stent complex with complete occlusion of the coiled jugular diverticulum.

A VIA 33 microcatheter was navigated over a Synchro 2 microguidewire into the right jugular bulb diverticulum. An 8 mm × 11 mm WEB SL intrasaccular flow disruptor was deployed without detachment in the diverticulum. Despite multiple repositioning, the wide neck configuration of the diverticulum precluded stable deployment of the WEB device. The WEB device and VIA 33 microcatheter were removed.

A Navien 072 intermediate catheter was advanced over a Headway 27 and Synchro 2 microwire into the right transverse venous sinus. The Headway 27 was navigated over the across the contralateral transverse sinus into the left jugular bulb. The Synchro 2 microwire was exchanged for a balanced heavy weight 0.014″ microwire. The wire was positioned into the posterior superior sagittal sinus. The AXS Infinity guide sheath was advanced into the mid sigmoid venous sinus. The intermediate catheter was removed. Then, a Carotid Wallstent stent was advanced over the wire and positioned into the distal right sigmoid venous sinus. The stent was deployed under fluoroscopic guidance. Angioplasty of the stent with 6 mm Emerge (Boston Scientific, Marlborough, MA) followed by 8 mm Sterling balloon resulted in excellent stent expansion spanning from the sigmoid sinus to the proximal jugular vein. Under fluoroscopic guidance, an SL-10 microcatheter (Stryker, Portage, MI) was advanced over Synchro 2 wire into right jugular bulb diverticulum for coiling of the JBD. Post-procedure angiography was performed demonstrating no evidence of residual flow into the right jugular bulb diverticulum (Figures 2b,c). Following the procedure, the patient had complete resolution of her PT.

## Discussion

We encountered 14 patients with PT suspected to be due to a JBD, of which 12 underwent endovascular embolization, and had significant improvement or resolution of their symptoms. Treatment was performed either with stent assisted coil embolization or placement of a WEB device. We routinely perform a BTO to assess PT improvement with JBD occlusion. Two patients were unable to hear their PT at the time of procedure (due to intermittent nature or positioning) and were not tested. All eight patients who underwent BTO showed at least mild improvement in PT We believe that BTO is an important part of the workup as it helps predict the outcome of JBD embolization. In three patients, saline was injected into the JBD as an additional test and resulted in recreation of their symptoms. To the author's knowledge, this is the largest series on the endovascular treatment of JBD.

As all 14 patients with JBD identified at our institution during the study period had debilitating PT, and response to BTO, reproduction of PT symptomology with provocative saline injection testing, or discussions around past failed conservative management prior to treatment decisions served as key differentiating factors between the 12 endovascularly treated patients and 2 conservatively managed patients, selection bias did not have a significant impact on treatment choice.

There is significant nuance to stenting across the jugular bulb. One recent series demonstrated a 33% rate of periprocedural complications including one case of stent migration into the heart (8). To address these challenges, our institutional practice is to initiate stent placement in the sigmoid sinus and then to subsequently either use one long braided stent (Carotid Wallstent) or two overlapping open cell stents (Precise Pro), relying on the open cell design to anchor the second stent in place (Figure 3). Balloon inflation is often to improve wall apposition of the stent across the Jugular Bulb, which also carries risk of stent migration; however, there were no cases of stent migration in our series. The other option pursued was to place a single WEB in the JBD and rely on the lateral compression of the device to maintain its placement (9). However, this is not always guaranteed as we did have one case of WEB migration post detachment which was seen during post procedure angiography. The WEB in this case was removed using a snare. In all cases of WEB placement, we utilized balloon reconstruction to ensure placement in the JBD and a normal venous channel for drainage. The WEB device is a mesh made up of nitinol wires and a platinum core that can self-expand within an aneurysm to lead to thrombosis of the aneurysmal area (10). Although this device is designed to be used to treat wide-neck intracranial arterial aneurysms (11), it has been reported in use in patients with JBD, such as in one case report studying the use of WEB in a patient with JBD and ipsilateral PT (12). Further study into the efficacy of WEB devices in the treatment of aneurysms of the jugular bulb would be beneficial, as two of the three WEB devices used in our cohort were removed due to suboptimal placement.

**Figure 3:**
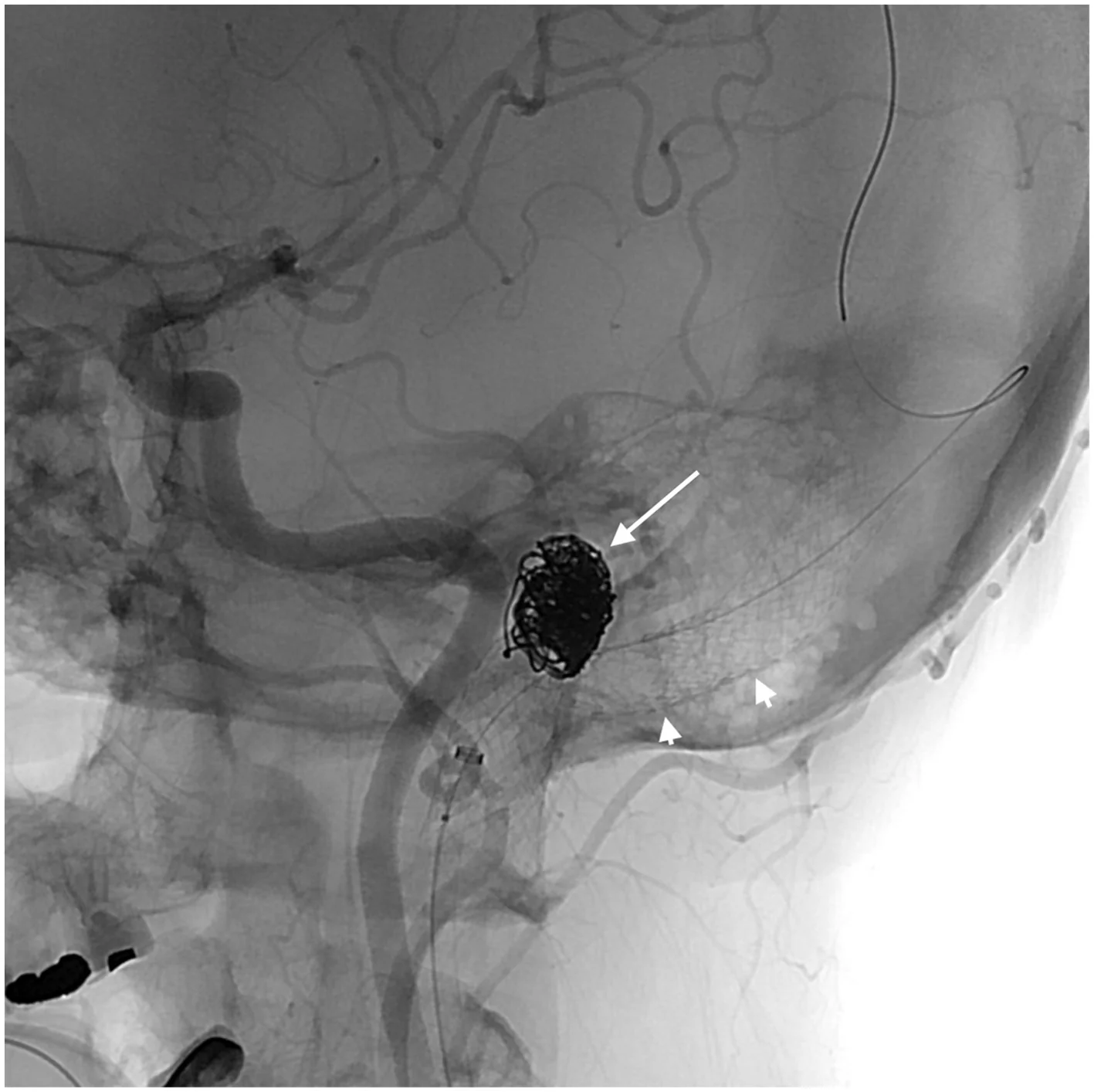
Unsubtracted lateral x-ray showing a coil mass within a right jugular bulb diverticulum (white arrow) and two overlapping open cell stents (overlapping portion within the sigmoid sinus indicated by white arrowheads), demonstrating how the open cell design anchors the sigmoid sinus-jugular bulb stent in place within the traverse sinus-sigmoid sinus stent.

JBD is a rare pathology with limited representation in the literature, and reports on its treatment are even more infrequent. To date, most cohorts studying endovascular treatment of JBD are either single patient case reports or small cohorts. However, with the development of new endovascular devices and techniques, endovascular treatment options for JBD patients experiencing PT have expanded. A study providing a case report and literature review identified five patients with JBD-−4 treated with stent-assisted coil embolization and 1 treated with stenting—who experienced no complications, and 80% maintained complete resolution of symptoms 1 year post-procedure (13). Additionally, in a single-center study where 7 JBD patients with PT were endovascularly treated, five out of seven (71.4%) of patients had partial or complete symptom resolution (14). Stent-assisted coil embolization in a patient with bilateral JBD in two separate procedures also resulted in successful resolution of symptoms 10 months post-procedure with no complications (9). A case report demonstrated the feasibility of WEB device placement in JBD, resulting in no post-procedural complications, complete resolution of PT 5 days post-intervention, and complete thrombosis of JBD without affecting flow in the jugular vein and sinus on 3-month angiography images (12). A case report involving stent-coiling embolization in a novel venous double-jailing technique with volumetric coils was used to treat two diverticula on the right jugular bulb simultaneously, resulting in immediate and persistent symptom resolution without any complications (15). Our slightly larger database's results contribute to the growing practice of endovascular neurointervention for JBD (9, 16, 17). Endovascular treatments are often necessary for PT because conservative treatments, such as acetazolamide (Diamox), typically fail to resolve patient symptoms over the long-term. Acetazolamide (Diamox) is used to reduce intracranial pressure through inhibition of carbonic anhydrase in the proximal convoluted tubule of the kidneys, leading to diuresis that can reduce overall CSF volume (18). Although acetazolamide (Diamox) may assist with reducing intracranial pressure, the side effects of this medication, including paresthesia of the mouth, fatigue, and malaise, can make adherence to the medication intolerable for patients, leading them to seek other therapeutic modalities to reduce symptoms such as PT (18). Surgical treatment options for JBD causing PT are sparse in the literature. One study reported two JBD causing PT being treated with transmastoid resurfacing, resulting in complete symptom cessation of symptoms post-operative, with no major intraoperative or postoperative complications (19). Another case report described a JBD causing PT being treated with hypotympanic resurfacing with hydroxyapatite cement, with the patient experiencing mild hyperacusis, but achieving complete symptom resolution at 33 months (20). Overall, endovascular treatments are minimally invasive with low complication rates while still achieving high symptom resolution rates compared to surgical treatment, and therefore are currently the preferred treatment option for PT caused by JBD.

The management of venous PT in the setting of transverse-sigmoid sinus pressure gradients has been well described in the literature with VSS demonstrating high rates of symptomatic resolution (4). Only one of our seven patients had a significant transverse-sigmoid sinus gradient of at least 7 mm Hg during venous sinus pressure testing. All other patients had no notable VSS on imaging or only had a gradient of 1 mm Hg, increasing our suspicion of an alternative source. As detailed above, the one patient with a gradient underwent VSS of the transverse sigmoid junction and woke up with no symptom improvement. She was taken immediately back and underwent awake WEB placement in the JBD which significantly improved her symptoms. JBD is a unique entity requiring very specific treatment considerations as demonstrated by the various techniques utilized in this series.

## Limitations and future directions

Our study includes twelve patients with JBD who underwent endovascular intervention between September 1, 2020 to April 30, 2026. Although our cohort is the largest current series of endovascular treatment of JBD, the total patient number is still limited. As more patients are recruited for neurointerventions, we hope to continue expanding our database of patients with PT caused by JBD to study the most effective combination of stenting, coils, angioplasty, and WEB use. Additionally, we hope to implement preventative strategies with our patients, such as BMI management, to reduce the risk of PT.

Minimally invasive strategies to treat rare conditions such as JBD are evolving and, with future studies, comparisons between surgical treatments and endovascular interventions should be undertaken. There are studies of surgical treatment for JBD in the literature that are preliminary, such as transmastoid reconstruction/resurfacing of the jugular bulb. Thus, such comparative studies between minimally invasive and invasive interventions can allow physicians to best choose a strategy to resolve such cases of PT (19).

## Conclusion

Endovascular treatment of JBD with stent-assisted coiling is safe and effective for the management of PT. BTO is an important intraprocedural tool that can be used to help predict treatment success. Future studies should investigate the best possible use, if any, of WEB devices for this pathology.

## Data Availability

The raw data supporting the conclusions of this article will be made available by the authors, without undue reservation.
